# Acceptance and Readiness for AI Among United Arab Emirates–Based Health Care Practitioners: Exploratory Cross-Sectional Survey

**DOI:** 10.2196/80173

**Published:** 2026-04-17

**Authors:** Ghufran Alsalloum, Yara Badr, Ayman Alzaatreh, Abdulrahim Shamayleh, Muhammad Kumail, Nour Aymn Ahmad, Yacine Hadjiat

**Affiliations:** 1 Bioscience and Bioengineering, College of Engineering, American University of Sharjah, University City, S120 University City St, Sharjah, 26666, United Arab Emirates, 971 553469390; 2 Department of Mathematics and Statistics, College of Arts and Sciences, American University of Sharjah, Sharjah, United Arab Emirates; 3 Department of Industrial Engineering, College of Engineering, American University of Sharjah, Sharjah, United Arab Emirates; 4 Dubai Health Innovations, Mohammed Bin Rashid University of Medicine and Health Sciences, Dubai, United Arab Emirates; 5 Department of Clinical Pharmacy and Pharmacology, College of Pharmacy, Ras al-Khaimah Medical and Health Sciences University, RAK, United Arab Emirates

**Keywords:** artificial intelligence, technology acceptance, trust, perceived risk, perceived benefit, readiness, structural equation modeling, United Arab Emirates, health workforce, survey methods

## Abstract

**Background:**

Artificial intelligence (AI) can enhance diagnostic accuracy, efficiency, and decision-making in health care, but real-world impact depends on practitioners’ acceptance and readiness to use AI in clinical workflows. The United Arab Emirates offers a policy-driven context to study these factors, given active national AI strategies and rapid health system digitization.

**Objective:**

This study aimed to develop and validate a model explaining how trust, perceptions, perceived risk, and perceived benefit shape practitioners’ acceptance of AI and, in turn, their readiness to implement AI in clinical practice. The model integrates the Technology Acceptance Model, the Unified Theory of Acceptance and Use of Technology, and the Theory of Trust and Acceptance of Artificial Intelligence Technology.

**Methods:**

We conducted a cross-sectional online survey of 182 United Arab Emirates–based health care practitioners (physicians, nurses, dentists, and allied health staff). Constructs included trust, perception, perceived risk, perceived benefit, acceptance, and readiness. Knowledge of AI was also assessed using true or false statements. We performed confirmatory factor analysis and structural equation modeling, reporting standard fit indices. The survey adhered to the Checklist for Reporting Results of Internet E-Surveys guidelines, and ethics approval and electronic consent were obtained.

**Results:**

Trust was positively associated with perception (*β*=.704; *P*<.001) and perceived benefit (*β*=.191; *P*=.02) and negatively associated with perceived risk (*β*=−.301; *P*<.001). Acceptance was positively associated with trust (*β*=.452; *P*<.001), perception (*β*=.459; *P*<.001), and perceived benefit (*β*=.168; *P*=.002), and negatively associated with perceived risk (*β*=−.140; *P*=.009). Acceptance strongly predicted readiness (*β*=.874; *P*<.001). The model fit indices are standardized root-mean-square residual of 0.068, root-mean-square error of approximation of 0.0913, goodness-of-fit index of 0.802, adjusted goodness-of-fit index of 0.763, and comparative fit index of 0.906. Our knowledge assessment found notable gaps among participants, underscoring a need for education and training. Our study sample was predominantly drawn from Dubai-based health care settings (103/182, 57%) and nursing roles (71/182, 39%); therefore, these findings primarily reflect the Dubai health regulatory environment and nursing workflows and may not generalize to the broader federal health care system across all Emirates.

**Conclusions:**

Trust is a central lever for advancing AI acceptance and implementation readiness among the study cohort of United Arab Emirates–based health care practitioners. Implementation programs should prioritize building institutional and technical trust (transparency, safety, and governance), reducing perceived risk (privacy, security, and reliability), and amplifying perceived benefits through hands-on demonstrations and workflow-aligned use cases. Targeted training to close knowledge gaps should accompany policy and organizational measures aligned with national AI strategies to accelerate responsible, clinician-in-the-loop adoption.

## Introduction

### Overview

The integration of artificial intelligence (AI) is instrumental in driving innovation and enhancing operational efficiencies across diverse sectors, ranging from finance and manufacturing to transportation and entertainment [1,2]. In health care, AI has emerged as a game changer, offering unprecedented opportunities to transform the delivery of medical services and improve patient outcomes [2,3]. The integration of AI in health care is expected to expand into various domains, including drug discovery, personalized medicine, and predictive analytics [4,5]. An important aspect of AI integration into health care is the readiness of health care practitioners to accept and use AI technology in their practice [6]. Various studies have attempted to understand the factors affecting the integration of AI into health care. While some studies focused on the perspectives of students [7-11], the focus of the current literature review is on the perspectives of health care professionals as the main players in the integration of AI in health care.

### Background

#### Overview

The integration of AI into health care practice represents a transformative shift that demands careful consideration of how medical professionals perceive, accept, and ultimately use these technologies. Recent empirical investigations have uncovered a complex interplay of factors influencing health care professionals’ readiness to adopt AI in clinical settings.

A recurring theme across multiple studies is the critical importance of perceived usefulness as a primary driver of AI acceptance. Lambert et al [12] identified this factor as present in virtually all investigations of health care AI adoption. When clinicians believe AI systems will genuinely improve clinical outcomes, enhance diagnostic accuracy, or increase efficiency, they demonstrate significantly greater willingness to incorporate these tools into their practice. Moreover, a study found that, once faced with a health crisis, higher perceived usefulness and perceived risk of disease (eg, COVID-19) have significant positive effects on the physicians’ intention to engage with digital health work such as volunteering [13].

Equally important are the technical characteristics of AI systems themselves. Explainability, transparency, and interpretability are foundational to building trust among health care professionals [14]. The perceived “black box” nature of many AI algorithms remains a significant barrier to widespread adoption, with clinicians expressing a reluctance to rely on systems whose decision-making processes they cannot fully understand or validate.

Workflow compatibility represents another crucial consideration. Multiple studies highlight that AI tools perceived as disruptive to established clinical workflows face substantial resistance, while those offering a seamless integration experience a more favorable reception. Hogg and Al-Zubaidy [15] specifically identified difficulties in workflow integration as a major barrier to implementation success.

#### Specialty-Specific Perspectives

Understanding the adoption of AI in health care requires attention to the unique perspectives and contextual factors within different medical specialties. This section examines how specialty-specific perspectives have been studied in the literature, offering nuanced insights into the facilitators and barriers encountered by diverse professional groups.

##### Radiologists and Radiographers

Radiology has emerged as a focal point for AI implementation research, likely due to the image-centric nature of the specialty and early AI successes in this domain. A large-scale cross-sectional study of 3666 radiology residents in China examined perceptions of AI replacement, usefulness, and acceptance [16]. The authors found that most residents held positive attitudes toward AI. Key predictors of AI acceptance included age, gender, education, and geographic region. Notably, those with prior AI experience were more likely to recognize its usefulness and support its adoption.

In an international survey involving 1041 radiologists and radiology residents across 54 countries, 2 complementary studies [17,18] explored AI-specific knowledge, fear of replacement, attitude, and expectations. The studies found that fear was associated with lower levels of AI knowledge**,** while positive attitudes correlated with more advanced knowledge [16]. Additionally, most respondents anticipated AI would play a supporting role (eg, second reader) in radiology within the next decade. A total of 79% of participants endorsed integrating AI into residency programs**,** particularly for training in data management and ethics [18].

These findings align with the observation by Shamszare and Choudhury [19] that health care professionals with AI experience often find systems challenging to learn initially, underscoring the importance of education and training. Indeed, only 17% of respondents in their study reported having used AI in clinical practice, highlighting significant adoption gaps. Similar trends emerged in a cross-sectional survey of 562 Saudi radiographers [20], which stated radiographers largely view AI as integral to the future of diagnostic imaging, although concerns about high costs, lack of technical expertise, and cybersecurity threats were prevalent. A broader African study involving 1020 radiographers explored attitudes toward AI in medical imaging [21]. The results indicated that participants believed AI would enhance quality assurance and radiographic practice. However, concerns around job security and professional displacement were prominent, especially among younger respondents.

##### Emergency and Surgical Settings

The acute, time-sensitive nature of emergency medicine and surgery creates unique considerations for AI adoption. A survey conducted among 113 members of the American Society of Emergency Radiology focused on AI implementation, governance, trust, and expectations in emergency radiology [22]. Most respondents reported using commercial AI tools and emphasized the need for transparent and explainable models to build trust and ensure accountability in emergency decision-making contexts. Another relevant study surveyed 650 surgeons affiliated with the World Society of Emergency Surgery across 71 countries [23]. The results revealed mixed attitudes, with a preference for traditional clinical decision-making tools over AI in many cases.

Complementing this, the Artificial Intelligence in Emergency and Trauma Surgery project involved 200 World Society of Emergency Surgery surgeons, using an online questionnaire to evaluate knowledge, expectations, and AI adoption [24]. A majority of participants (74.5%) expressed confidence that AI would soon be implemented in their settings. However, only 38.5% actively read AI-related literature, revealing a gap between interest and informed engagement. These findings echo concerns identified by Khanijahani et al [25] regarding professional autonomy. Many health care professionals, particularly in high-stakes environments, worry that AI may undermine clinical judgment or potentially replace professional roles, concerns that appear more pronounced among more experienced practitioners.

##### Broader Medical Workforce Perspectives

A study assessing AI perceptions among both 1516 health care workers and 1264 non–health care workers in China [26] examined receptivity, safety perception, and demand for medical AI. Both groups demonstrated high levels of receptivity, although health care workers showed a more nuanced understanding of safety and impact concerns. A smaller study involving 77 medical doctors from 13 specialties assessed ethical perspectives on health AI [27]. The study identified 4 distinct viewpoints, including those who view AI as a helpful tool and others who emphasize the need for explainable AI and concern over private sector involvement.

A qualitative study of 22 United Kingdom National Health Service professionals [28] explored perceived usefulness and ease of use using semistructured interviews. Participants included trauma surgeons, general practitioners, and medical educators. The results revealed that perceived usefulness was associated with improved efficiency, care quality, and diagnostic accuracy. Perceived ease of use was challenged by compatibility issues, complexity, ethical concerns, and training needs. These findings highlight that positioning AI as a supportive tool rather than a replacement enhances acceptability. Finally, a mixed cohort study involving 105 medical doctors and 102 medical students assessed familiarity with AI, education, risks, and implementation challenges [29]. The study found no significant difference in AI familiarity between the two groups, although students reported higher perceived risks. Both groups reported low participation in formal AI training, reinforcing the finding by Chowdhury et al [30] that targeted education and training significantly improve knowledge, attitudes, and readiness for AI adoption.

### AI in UAE Health Care

The United Arab Emirates presents an interesting case study in AI adoption across health care contexts. AI adoption in the United Arab Emirates is a topic of growing interest across various sectors. Research has explored AI’s impact on different fields in the United Arab Emirates, such as air quality monitoring [31], journalism practices [32], e-innovative projects in the public sector [33], and the legal framework surrounding AI decisions and explanations [34]. These studies highlight the importance of understanding AI mechanisms, addressing biases, and establishing ethical guidelines to ensure responsible AI use.

In the dental domain, a cross-sectional exploratory study involving 134 participants (including 72 undergraduate dental students, 19 academic staff, and 44 practicing dentists) investigated perceptions, knowledge, and organizational readiness for AI implementation [35]. Using structured questionnaires, the study found that the majority of participants had medium to high knowledge of AI, particularly among female students. Significant associations were observed between demographics and AI perceptions, underscoring the role of background characteristics in shaping adoption readiness. In the radiology domain, a cross-sectional study of 153 radiology professionals (radiologists and radiographers) in UAE hospitals explored knowledge, perceptions, readiness, and challenges regarding AI integration [36]. The findings revealed a lack of AI awareness, particularly among radiographers. While there was some interest in AI for postprocessing and dose management, most participants were unaware of AI’s broader potential in radiology. The study concluded that AI literacy remains low, with wide disagreement about AI’s importance in clinical radiology.

Using partial least squares structural equation modeling (SEM), a study with 53 participants (from IT and health departments) identified the critical success factors for AI project implementation in the UAE public health sector [37,38]. These included managerial, operational, organizational, IT infrastructure, and strategic factors. All were found to be significantly associated with both the perceived usefulness and ease of use of AI technologies. The study emphasizes that structural and institutional support are key to ensuring AI adoption success, making these variables crucial candidates for inclusion in SEM. Another large-scale study of 553 nurses across the United Arab Emirates assessed knowledge, perception, and organizational readiness toward AI in nursing [39]. Using descriptive statistics and chi-square tests, the study revealed that perceptions of AI varied significantly by age, educational background, and work experience. Notably, older and more experienced nurses reported feeling more threatened by AI, suggesting an age-related barrier that could influence trust and perceived risk in SEM models. Additionally, an analytic hierarchy process–based study conducted with 27 health care executives in the United Arab Emirates evaluated factors affecting AI adoption, including concerns over accuracy, data security, and privacy [40]. While this study is not open access, it contributes to the understanding that managerial perceptions often prioritize ethical and infrastructural safeguards, which are essential for implementation readiness.

### Scope of the Current Study

This study aims to develop and validate a comprehensive model of factors associated with health care professionals’ acceptance and readiness to adopt AI in clinical practice within the UAE health care system. By spanning diverse professional groups, using advanced analytical techniques, and integrating multiple theoretical frameworks, this study aims to provide actionable insights for health care leaders, policymakers, technology developers, and educators seeking to facilitate successful AI implementation in health care settings.

This study makes a significant contribution to the literature by specifically examining AI adoption within the UAE health care context. The United Arab Emirates presents a unique research setting characterized by rapid technological advancement, substantial investment in health care infrastructure, and a highly diverse health care workforce representing various cultural and educational backgrounds. Understanding AI adoption patterns in this context has particular relevance given the “We the UAE 2031” vision [41] and the National Strategy for Artificial Intelligence 2031 [42] and its emphasis on developing world-class health care infrastructure supported by advanced technologies. The findings from this research will directly inform implementation strategies aligned with these national priorities.

Despite rapid national AI initiatives, little is known about front-line practitioners’ acceptance and readiness in the United Arab Emirates. This study addresses that gap using a model based on the Technology Acceptance [43], the Unified Theory of Acceptance and Use of Technology (UTAUT) [44], and the Theory of Trust and Acceptance of Artificial Intelligence Technology (TrAAIT) [45] models.

## Methods

### Study Design

**Figure 1.**
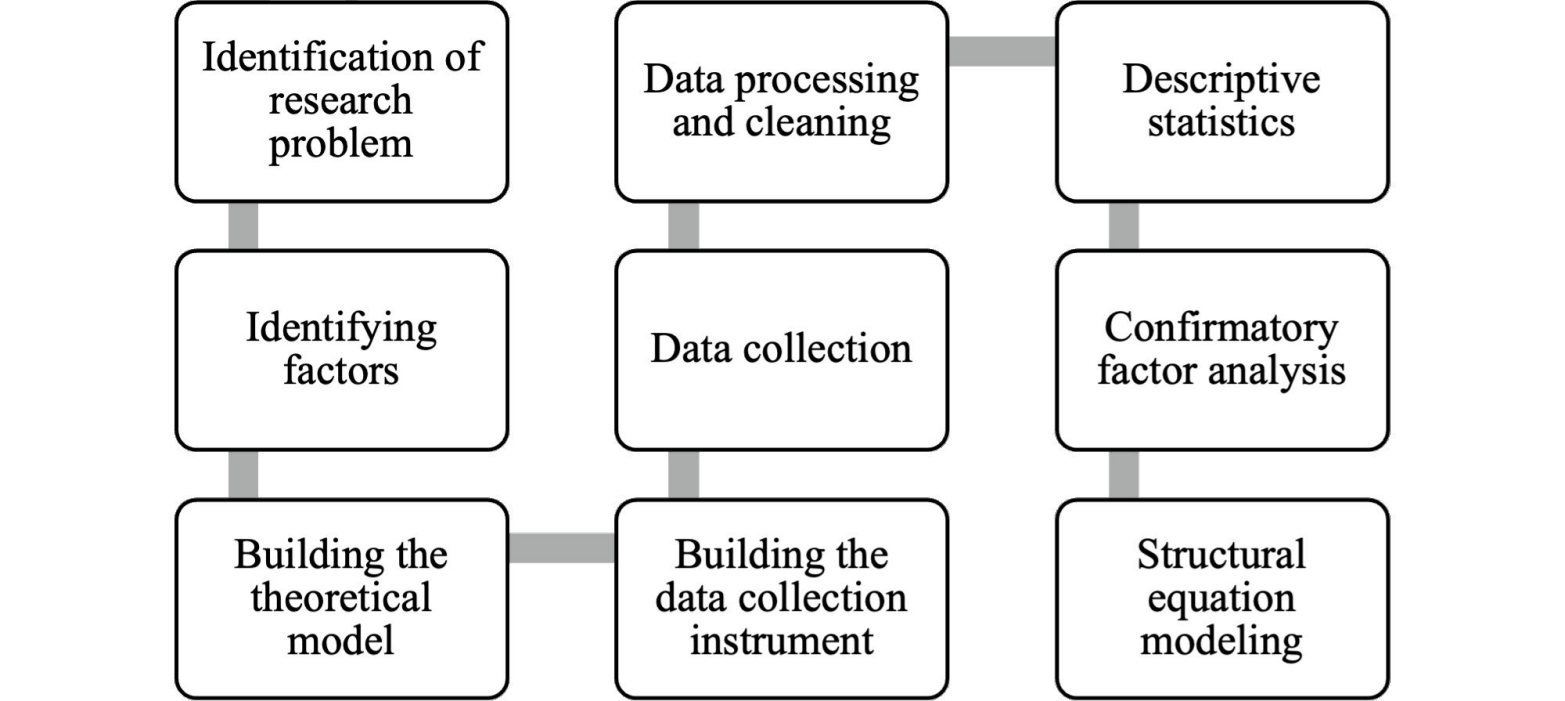
Study flow and analysis plan for a cross-sectional online survey of United Arab Emirates–based health care practitioners. The diagram shows recruitment, measures, and analysis steps (reliability and validity testing, confirmatory factor analysis, and structural equation modeling).

This study adhered to the Checklist for Reporting Results of Internet E-Surveys (CHERRIES) for web-based survey research [46] (Checklist 1). A cross-sectional online survey was designed based on the Technology Acceptance [43], UTAUT [44], and TrAAIT [45] models. Ethical approval was obtained, and informed consent was secured electronically. The instrument was pretested and refined before full deployment, using validated constructs. Participants signed an electronic informed consent through a tick box, and the survey was deployed on a web-based platform accessible via desktop and mobile. Since the survey was shared through the professional network of the authors, the response rate could not be precisely calculated. To ensure data integrity, duplicate entries were prevented using cookies and IP filtering, and the survey form was configured to accept only fully completed responses. For analysis, confirmatory factor analysis (CFA) and SEM were performed using the SAS (SAS Institute) software [47]. The study acknowledges limitations related to potential self-selection and response bias. The methodology used in this study consists of sequential steps, as demonstrated in Figure 1.

### Theoretical Model

#### Overview

Synthesizing findings across the literature, several consistent themes emerge regarding health care professionals’ attitudes toward AI technologies (Table 1). Six constructs were extracted from the literature: Trust, Perception, Risk, Benefit, Acceptance, and Readiness. These constructs were selected based on their conceptual clarity, recurrence across research, and demonstrated predictive validity in AI adoption behavior. We ground acceptance in the Technology Acceptance Model (TAM), operationalizing it as a higher-order construct centered on perceived usefulness, perceived ease of use, attitude, and intention as adapted for AI contexts [48]. Perception follows the Shinners Artificial Intelligence Perception instrument’s two subdomains, perceived professional impact, and preparedness for AI, to capture clinicians’ overarching stance toward AI [49]. Our Trust construct draws on a UTAUT and TrAAIT-based model in risk analysis, showing how comparative trust in AI versus clinicians channels through Risk and Benefit appraisals to shape preferences, which we leverage to position trust as an upstream driver of Risk and Benefit assessments [50]. Readiness is measured with the medical AI readiness scale for medical students, encompassing cognition, ability, vision, and ethics, to reflect deployable capacity rather than mere intention [51]. Our integrated framework adds value by linking beliefs to intention and capacity. This gives a governance-aware mechanism rather than attitudes alone. Using validated, domain-specific instruments improves content validity and comparability, and separating Perception from Acceptance disentangles overlapping drivers. Building on the reviewed literature, the proposed SEM model is not only theoretically robust but also empirically grounded. The paths from trust to intermediate beliefs, from those beliefs to Acceptance, and from Acceptance to Readiness are explained below.

**Table 1.** Summary of constructs from TAM^a^, UTAUT^b^, and TrAAIT^c^ that are used to design the instrument, with item counts and sources.

Sources	Perception	Trust	Risk	Benefit	Acceptance	Readiness	Knowledge
Cho and Seo [7]	✓		✓		✓		
Chen et al [9]					✓		
Caparrós Galán and Sendra Portero [10]	✓						
Truong et al [11]	✓						✓
Lambert et al [12]		✓			✓		
Tucci et al [14]		✓					
Chen et al [16]	✓		✓	✓	✓		✓
Huisman et al [17] and Huisman et al [18]	✓		✓			✓	✓
Shamszare and Choudhury [19]	✓	✓	✓	✓			
Aldhafeeri [20]	✓		✓	✓			
Botwe et al [21]	✓		✓	✓			
Agrawal et al [22]	✓	✓	✓	✓	✓		
Cobianchi et al [23]	✓		✓	✓			
De Simone et al [24]	✓						✓
Xiang et al [26]	✓	✓	✓				
Martinho et al [27]	✓						
Hercheui and Mech [28]	✓		✓				
Boillat et al [29]						✓	✓
Chowdhury et al [30]	✓		✓	✓			
Hamd et al [35]	✓					✓	✓
Abuzaid et al [36]	✓		✓			✓	
Alhashmi et al [37] and Fs et al [38]	✓						
Abuzaid et al [39]	✓		✓			✓	
Stevens and Stetson [45]		✓			✓		
Sohn and Kwon [48]					✓		
Shinners et al [49]	✓						
Kerstan et al [50]		✓	✓	✓			
Karaca et al [51]						✓	✓
Ratta et al [52]		✓	✓		✓		
Wang and Wang [53]		✓			✓		
Castagno and Khalifa [54]	✓						
Ramot and Tal [55]		✓	✓	✓			

a TAM: Technology Acceptance Model.

b UTAUT: Unified Theory of Acceptance and Use of Technology.

c TrAAIT: Theory of Trust and Acceptance of Artificial Intelligence Technology.

#### Trust as an Exogenous Driver of Acceptance, Perception, Risk, and Benefit

The hypothesized SEM integrates evidence-based constructs and pathways to explain health care professionals’ acceptance and readiness for AI adoption. The model positions Trust as an exogenous latent variable that is linked to evaluations of Perception, Risk, and Benefit, which subsequently predict Acceptance. Readiness is treated as a downstream institutional outcome of individual acceptance. This arrangement is grounded in established trust and technology acceptance literature, including models validated within both general information systems and health care–specific contexts.

The model’s configuration, placing trust at the top, is supported by Mcknight et al [56], who conceptualize trust in technology as a foundational belief composed of perceptions about functionality, reliability, and helpfulness. This form of trust precedes evaluations such as usefulness or risk and serves as a precondition for deeper behavioral commitment. Gefen et al [57] further reinforce this positioning by demonstrating that trust, while distinct from perceived usefulness and ease of use, significantly links to both and has a direct link to behavioral intention to use technology. In clinical settings, Stevens and Stetson [45] validated trust as the strongest determinant of AI acceptance among clinicians, accounting for more than half of the explained variance in their model. These findings consistently support the treatment of trust as a higher-order, exogenous factor in models of technology adoption.

#### Trust Associated With Acceptance, Perception, Risk, and Benefit

Trust is well-established in the literature as a primary determinant of downstream evaluations in technology adoption contexts. Bahari et al [58] found that trust significantly reduced perceived risk and enhanced perceptions of benefit in a telemedicine and assistive technology context. Their study concluded that individuals with higher levels of trust were more likely to assess the technology positively and perceive fewer associated risks. Similarly, Alshehri et al [59], in their systematic review of the Internet of Medical Things adoption, observed that trust shaped expectations of usefulness while also moderating privacy-related concerns. Ratta et al [52] further validated these effects in an extended UTAUT model specific to health care AI, showing that trust is linked to perceived usefulness, risk appraisal, and acceptance intentions. Wang and Wang [53] analyzed trust factors in AI-assisted diagnosis within chronic disease management and found that trust significantly predicted doctors’ acceptance of AI systems. Their findings emphasized that higher trust, driven by factors such as transparency and interpretability, enhanced willingness to adopt AI tools. Collectively, these studies support the theorized Trust → Acceptance, Trust → Perception, Trust → Risk, and Trust → Benefit pathways.

#### Knowledge Associated With Perception, Risk, and Benefit

The relationship between knowledge and the perception of AI’s risks and benefits is well-supported in the literature, primarily through the knowledge deficit model. Although heavily criticized, the model posits that individuals with greater objective knowledge about scientific or technological innovations are more likely to assess their risks and benefits accurately [60]. In the context of AI in health care, this suggests that increasing factual knowledge reduces irrational fears and enhances recognition of AI’s benefits, while lowering perceived risks [50]. Several empirical studies have validated this relationship. Roy [61] found that physicians with higher levels of AI-related knowledge were more likely to perceive the technology as beneficial and less risky in the context of diabetes diagnostics. Similarly, other studies demonstrated that knowledge of IT is significantly linked to perceptions of clinical IT in the extended TAM and UTAUT models [62,63]. Additionally, Bach and Männikkö [64] highlighted how knowledge about AI in mental health care alters perception of benefit and risk, particularly in sensitive clinical environments. Together, these findings confirm that knowledge is significantly linked to the downstream constructs of perception, risk appraisal, and benefit recognition, supporting the integration of the Knowledge → Perception, Knowledge → Risk, and Knowledge → Benefit pathways within the SEM.

#### Perception, Risk, and Benefit as Predictors of Acceptance

The model’s assumption that perception, risk, and benefit function as direct antecedents to acceptance is similarly reinforced. Gerlich [65] found that perceived benefit and risk were both significant predictors of acceptance of AI tools in clinical settings. Notably, this study highlighted that the impact of perceived benefit on acceptance could override the dampening effects of risk, particularly when trust was present. Ramot and Tal [55] provided further evidence of these mechanisms in a telehealth context, showing that the perception of risk is negatively associated with acceptance, whereas perceptions of benefit served as a motivational driver. Maulana et al [66] supported this framework by demonstrating that perceived usefulness and data security concerns were significant predictors of adoption in mobile health apps. These findings offer clear empirical support for the Perception → Acceptance, Risk → Acceptance, and Benefit → Acceptance pathways.

#### Acceptance as a Predictor of Readiness

In this model, individual readiness is defined as the health care professional’s preparedness, across knowledge, skills, and attitudes, to apply AI technologies in clinical care. The pathway from Acceptance to Readiness is justified by the premise that behavioral intention (Acceptance) acts as a motivational antecedent to capacity-building behaviors. Once a health care professional accepts the relevance and use of AI, they are more likely to engage in knowledge acquisition, skill development, and ethical reflection behaviors that align with the dimensions of Readiness. Karaca et al [51] emphasize that students with a favorable attitude toward AI are more prepared to adopt AI systems cognitively and behaviorally. They further argue that AI readiness does not emerge in isolation but is dependent on prior acceptance of AI’s role in medicine and its alignment with professional identity.

Therefore, the following hypotheses are to be tested:

1. Hypothesis 1: Trust is positively associated with health care practitioners’ perception of AI.
2. Hypothesis 2: Trust is positively associated with health care practitioners’ perceived benefit of AI.
3. Hypothesis 3: Trust is negatively associated with health care practitioners’ perceived risk of AI.
4. Hypothesis 4: Trust is positively associated with health care practitioners’ acceptance of AI.
5. Hypothesis 5: Perception is positively associated with health care practitioners’ acceptance of AI.
6. Hypothesis 6: Perceived benefit is positively associated with health care practitioners’ acceptance of AI.
7. Hypothesis 7: Perceived risk is negatively associated with health care practitioners’ acceptance of AI.
8. Hypothesis 8: Acceptance is positively associated with health care practitioners’ readiness to adopt AI.

### Data Collection

#### Overview

Based on the proposed model, the survey assessed the following constructs: Trust, Perception, Risk, Benefit, Acceptance, and Readiness. Each construct was measured through some indicators, and each indicator was represented by one question in the survey using a 5-point Likert scale: (1) strongly disagree, (2) disagree, (3) neutral, (4) agree, and (5) strongly agree. The survey was anonymous and took approximately 10 minutes to complete. All survey responses were recorded, coded, and analyzed. The survey was piloted for validation purposes, and the questions were refined using feedback from a group of health care professionals. The definition of AI was stated at the beginning of the survey to familiarize respondents who had no previous knowledge. The survey was distributed online from April to December 2024, targeting health care professionals from all specialties and practices.

#### Sampling Method and Participants

The purposive sampling technique was used in this study for data collection. The participants were health care professionals working in various capacities within the United Arab Emirates health care system, including doctors, nurses, pharmacists, dentists, and allied health staff. Participants were recruited from the professional networks of the research team members by direct contact. Participant responses were anonymous and unidentifiable.

#### Data Collection Procedure and Instrument

The participants were provided with detailed information about the study’s purpose, the voluntary nature of their participation, and assurances of confidentiality. Participants were then invited to complete an online survey (jotform.com), which was developed based on existing literature and validated scales. The indicators were assessed and deduplicated, resulting in 36 indicators. No incentive was provided. The survey aimed to identify criteria for readiness to adopt AI-based technologies in health care by analyzing factors associated with the adoption of AI. The initial portion of the questionnaire comprised demographic questions. A knowledge assessment section was included with True or False statements (objective knowledge) and a question assessing the perceived level of knowledge of participants (subjective awareness). Knowledge was not included in the SEM model due to a lack of reliable, prevalidated measures. The second portion of the instrument used a Likert scale of the latent variables. The components and sources of the survey are shown in Table 2.

**Table 2.** Survey structure mapping factors and the 36 indicators assessed in the survey, with the source. The number of initial indicators per factor is as follows: Perception (3 indicators), Trust (5 indicators), Risk (6 indicators), Benefit (6 indicators), Knowledge (5 indicators), Acceptance (7 indicators), and Readiness (4 indicators). Some factors and indicators included in the survey were removed from later analysis due to poor performance.

Factors	Dimensions	Reference
Perception	Perception of professional impact (2 items) Perception of preparedness for AI^a^	[49]
Trust	Propensity to trust technologies Technical competence Reliability User autonomy Faith	[50]
Risk and benefit assessment	Risks (6 items) Benefits (6 items)	[50]
Knowledge	Subjective awareness (self-reported) Objective knowledge (tested; 4 items)	[50,54]
Acceptance	Perceived ease of use (2 items) Perceived usefulness Subjective norms or social influence Perceived behavioral control Enjoyment Behavioral intention	[48]
Readiness	Cognition Ability Vision Ethics	[51]

a AI: artificial intelligence.

#### Pilot Study

A pilot study was conducted with 10 participants, separate from those included in the main analysis. Feedback from this pilot phase prompted adjustments to the survey. Specifically, changes were made to the survey length to ensure it was concise and manageable, reducing the time required to complete it to approximately 10 minutes. Furthermore, the format of knowledge questions was modified to “true, false, or I do not know” for clarity and ease of response. These refinements aimed to enhance the survey’s feasibility and effectiveness in gathering relevant data from health care practitioners.

### Data Validation

This is an initial step before starting the analysis, as it tests the reliability and validity of the collected data. The internal consistency of each dimension was measured using Cronbach α and composite reliability (CR). In addition, the sample size adequacy for SEM was conducted using the Kaiser-Meyer-Olkin test [67]. A Kaiser-Meyer-Olkin value between 0.7 and 1 indicates that the sample is adequate. Any variables that reduced the value of α will be removed from subsequent analysis. The average variance extracted (AVE) quantifies the amount of variance in indicators that is explained by the underlying latent construct. A higher AVE value indicates that the indicators are strongly related to the construct and explain a substantial portion of their variance, suggesting good convergent validity. A value of 0.5 or higher is deemed an acceptable level of AVE. Discriminant validity was evaluated using the Heterotrait-Monotrait criterion, using <0.85 as a conservative cutoff or <0.90 as a more permissive cutoff. Because this study used a cross-sectional, self-reported questionnaire to measure both predictors and outcomes, we evaluated the potential for common method bias. We applied the Harman single-factor test and used the conventional decision rule that common method bias is unlikely to be a dominant concern when the first factor explains <50% of the total variance.

### Data Analysis

A CFA was conducted to validate the measurement model. This step confirmed that the observed survey items were statistically aligned with their corresponding latent constructs, including Perception, Trust, Risk, Benefit, Acceptance, and Readiness. The CFA demonstrated strong factor loadings and good model fit, supporting construct validity. Following this, SEM was applied to test the relationships among these validated constructs. The model evaluated how Trust, Benefit, Risk, and Perception are linked to Acceptance, and how Acceptance, in turn, is associated with Readiness to adopt AI technologies among health care professionals.

### Ethical Considerations

This study received ethics approvals from the American University of Sharjah Institutional Review Board (protocol number 24‐077) and from Dubai Health/MBRU (MBRU IRB-2024‐491). Participants reviewed an online information sheet and provided electronic informed consent before accessing the survey. No compensation or incentives were offered. No direct identifiers were collected; analytic data were deidentified before analysis and stored on access-controlled systems. No individual is identifiable in this paper.

## Results

### Overview

This section presents the empirical findings from the analysis of survey responses collected from health care practitioners regarding their attitudes and readiness toward AI integration.

### Descriptive Statistics

The study included a total of 182 health care practitioners from different specialties and practice settings across the United Arab Emirates. The demographics of the studied sample are shown in Table 3. These demographics reflect a predominantly female and relatively young group with diverse professional roles and varying levels of experience. Purposive, network-based recruitment may oversample digitally engaged professionals and certain Emirates. We report the full profile and restrict inference to the cohort. The majority of respondents lack AI training, and a substantial proportion are uncertain about recognizing AI in software, highlighting areas for potential educational interventions and professional development. Nurses comprised around 39% (71/182) of respondents, potentially skewing attitudes toward nursing workflows.

**Table 3.** Characteristics of survey respondents (eg, gender, education, role, emirate, experience, and age) in the United Arab Emirates during the study period from April to December 2024 (N=182).

Demographic category	Values
Gender, n (%)	
Woman	116 (63.74)
Man	66 (36.26)
Highest level of education obtained, n (%)	
Bachelor’s	113 (62.09)
Master’s	52 (28.57)
Doctorate	17 (9.34)
Current professional role, n (%)	
Nurse	71 (39.01)
Medical doctor	33 (18.13)
Dentist	26 (14.29)
Pharmacist	25 (13.74)
Other allied health forces	25 (13.74)
Laboratory specialist	2 (1.10)
Years of experience in health care (year), n (%)	
1‐5	44 (24.18)
6‐10	36 (19.78)
11‐15	34 (18.68)
16‐20	31 (17.03)
21+	37 (20.33)
Do you have prior experience or training in AI^a^ or machine learning?, n (%)	
No	137 (75.27)
Yes	45 (24.73)
Can you recognize when AI is used in a software?, n (%)	
Yes	77 (42.31)
Not sure	65 (35.71)
No	40 (21.98)
Emirate of practice, n (%)	
Dubai	103 (56.59)
Ras Al Khaima	31 (17.03)
Abu Dhabi	26 (14.29)
Ajman	13 (7.14)
Sharjah	6 (3.30)
Fujairah	3 (1.65)
Age group (years), n (%)	
20‐30	47 (25.82)
31‐40	63 (34.62)
41‐50	47 (25.82)
51+	25 (13.74)

a AI: artificial intelligence.

After the initial demographic inquiries, participants engaged in questions aimed at assessing their knowledge about AI (Table 4). Despite 49% (roughly half; 89/182) of participants indicating a high level of confidence (agree n=58/182 and strongly agree 31/182) in their AI knowledge, the objective measurement questions unveiled a contrasting result. While approximately half (n=99/182) of the respondents answered the first question correctly, out of 182, only 60 (32%), 31 (17%), and 20 (11%) answered the second, third, and fourth questions correctly, respectively.

**Table 4.** Participant responses to the knowledge assessment section in the survey, with one statement for subjective knowledge appraisal and 4 statements for an AI^a^ fact check.

Statements and response option	Values
I am well-informed about AI, n (%)	
Strongly disagree (1)	3 (1.65)
Disagree (2)	36 (19.78)
Neutral (3)	54 (29.67)
Agree (4)	58 (31.87)
Strongly agree (5)	31 (17.03)
Deep learning uses artificial neural networks with multiple layers, n (%)	
True (correct answer)	99 (54.40)
False	8 (4.40)
I do not know	75 (41.21)
Unsupervised machine learning methods make use of training cases with labeled data, n (%)	
True	70 (38.46)
False (correct answer)	60 (32.97)
I do not know	52 (28.57)
Al-based outputs are free of biases, n (%)	
True	74 (40.66)
False (correct answer)	31 (17.03)
I do not know	77 (42.31)
At its core, Al relies on decision rules that are predefined by humans, n (%)	
True	121 (66.48)
False (correct answer)	20 (10.99)
I do not know	41 (22.53)

a AI: artificial intelligence.

### Data Validation

We assessed internal consistency, composite reliability, and convergent validity via AVE. Thresholds follow standard psychometrics or SEM texts. For the sample in this paper, the Kaiser-Meyer-Olkin value was 0.883, which implies that the sample was adequate. It is worth mentioning that the variable Benefit 1 reduced the value of α and was removed from the analysis. Cronbach α and CR values for most factors are approximately between 0.67 and 0.92, which are consistent with the acceptable level of 0.7. Although the Perception construct shows a marginal Cronbach α value of 0.67, it has been retained because all standardized indicator loadings exceed the acceptable level of 0.5. However, the Knowledge factor showed low reliability with α and CR equal to 0.14 and 0.42, respectively. Therefore, the Knowledge factor was excluded from the subsequent analysis.

Although perception (AVE=0.41) and risk (AVE=0.42) fall below the 0.50 benchmark, both factors show adequate internal consistency and composite reliability (Perception: *α*=.67, CR=0.67; Risk: *α*=.81, CR=0.81) with most loadings≥0.50. According to Fornell and Larcker [68], AVE is a conservative index of convergent validity, and a construct with AVE<0.5 may be retained when it has an acceptable level of reliability. Accordingly, these two factors were kept in the final model. The reliability and validity measures for the collected data are demonstrated in Table 5 (with the CFA results).

**Table 5.** Reliability and validity statistics (Cronbach α, CR^a^, AVE^b^) and CFA^c^ loadings and *P* value by construct in the SEM^d^. At least 3 indicators for each factor were retained.

Factors and indicators	Correlation with the total	CFA standardized parameter estimates	CFA unstandardized parameter (*P* value)	Cronbach α	CR	AVE
Perception				0.666	0.671	0.408
Perception 1	0.422	0.523	<.001			
Perception 2	0.526	0.701	<.001			
Perception 3	0.485	0.677	<.001			
Trust				0.917	0.919	0.696
trust 1	0.721	0.776	<.001			
trust 2	0.853	0.897	<.001			
trust 3	0.834	0.867	<.001			
trust 4	0.819	0.873	<.001			
trust 5	0.710	0.748	<.001			
Acceptance				0.912	0.903	0.572
acceptance 1	0.668	0.636	<.001			
acceptance 2	0.706	0.686	<.001			
acceptance 3	0.810	0.832	<.001			
acceptance 4	0.686	0.797	<.001			
acceptance 5	0.686	0.708	<.001			
acceptance 6	0.672	0.805	<.001			
acceptance 7	0.821	0.806	<.001			
Readiness				0.871	0.871	0.627
readiness 1	0.711	0.813	<.001			
readiness 2	0.768	0.833	<.001			
readiness 3	0.701	0.738	<.001			
readiness 4	0.714	0.780	<.001			
Risk				0.806	0.81	0.419
risk 1	0.462	0.519	<.001			
risk 2	0.521	0.607	<.001			
risk 3	0.546	0.594	<.001			
risk 4	0.611	0.685	<.001			
risk 5	0.700	0.800	<.001			
risk 6	0.550	0.644	<.001			
Benefit				0.865	0.863	0.561
benefit 2	0.669	0.694	<.001			
benefit 3	0.778	0.809	<.001			
benefit 4	0.792	0.894	<.001			
benefit 5	0.564	0.632	<.001			
benefit 6	0.635	0.685	<.001			
Knowledge				0.140	0.418	0.204
knowledge 1	0.039	0.923	<.001			
knowledge 2	0.048	0.231	<.001			
knowledge 3	0.085	0.284	<.001			
knowledge 4	0.063	0.115	0.04			
knowledge 5	0.068	0.137	0.01			

a CR: composite reliability.

b AVE: average variance extracted.

c CFA: confirmatory factor analysis.

d SEM: structural equation modeling.

Discriminant validity was assessed using the heterotrait-monotrait ratio (HTMT). All interconstruct HTMT values were below the conservative threshold of 0.85, supporting discriminant validity across the measurement model. HTMT matrix is reported in Table 6. Overall, the HTMT results suggest that the latent variables represent distinct constructs rather than redundant measurements of a single underlying factor. The Knowledge construct is not included in the table, as it is excluded from subsequent analysis (see below).

**Table 6.** HTMT^a^ discriminant validity matrix showing that all construct pairs are below 0.85, supporting discriminant validity. The highest overlap is Acceptance-Readiness (0.848), meaning they are strongly related but still distinct. Other relatively high (but acceptable) pairs are Perception-Acceptance (0.835) and Trust-Acceptance (0.832).

	Trust	Risk	Benefit	Perception	Acceptance
Trust					
Risk	0.309				
Benefit	0.175	0.207			
Perception	0.729	0.371	0.23		
Acceptance	0.832	0.381	0.33	0.835	
Readiness	0.812	0.305	0.203	0.773	0.848

a HTMT: heterotrait-monotrait ratio.

Harman single-factor test indicated that the first unrotated factor had an eigenvalue of 10.9480 and accounted for 36.49% of the total variance, which is below the 50% benchmark. This suggests that a single common method factor is unlikely to be the primary driver of the observed associations among constructs in this dataset.

### About CFA

In this step, CFA was conducted on the covariance matrix in order to validate the measurement model. The CFA results are shown in Table 5. All indicators were significant (*P* <.05) with acceptable loading sizes (standardized parameter estimates). Across constructs, internal consistency ranged from excellent to borderline, with the knowledge construct failing. Trust, Acceptance, Readiness, and Benefit demonstrated strong reliability. By contrast, Knowledge exhibited inadequate reliability and was excluded from the SEM. The goodness-of-fit indices (GFIs) for the CFA model are as follows: standardized root-mean-square residual (SRMR)=0.0680, root-mean-square error of approximation (RMSEA)=0.0913, GFI=0.8022, adjusted goodness-of-fit index (AGFI)=0.7629, and Bentler comparative fit index (CFI)=0.9058. The SRMR value falls within the acceptable threshold of less than 0.08. The RMSEA value exceeds the acceptable upper limit of 0.05. The CFI falls within the acceptable level of greater than 0.9. Both GFI and AGFI are less than the acceptable level of 0.85. Although the SRMR and CFI met commonly cited thresholds, the RMSEA and GFI fall outside conventional cutoffs for good model fit, indicating moderate to mediocre overall fit of the proposed model to the observed data [69].

### About SEM

**Figure 2.**
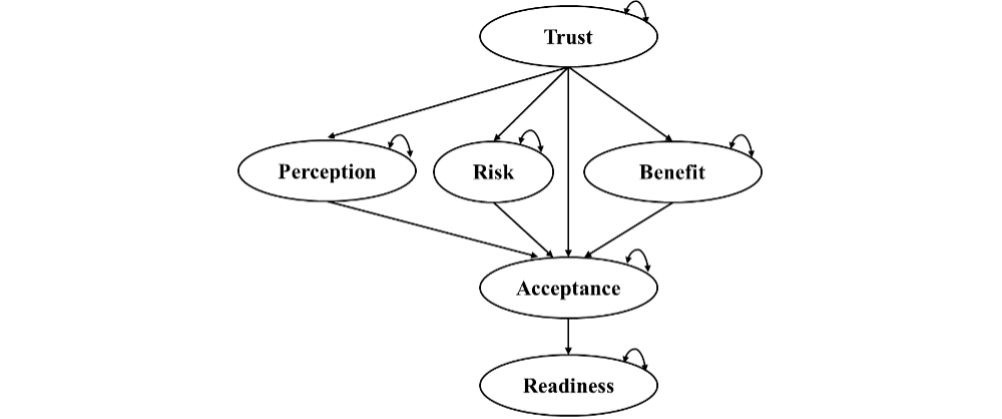
Final structural equation modeling of factors associated with acceptance and readiness for artificial intelligence with standardized path coefficients.

The theoretical model that reflects the hypotheses 1 to 8 is depicted in Figure 2. PROC (procedure) covariance analysis of linear structural equations in SAS software [47] is used to fit the model to the data. The results of the structural model are depicted in Table 7. Based on the results, all Hypotheses 1 to 8 are significant. First, the health care practitioners’ perception and perceived benefit are positively associated with trust (Hypotheses 1 and 2). While perceived risk is negatively associated with trust (Hypothesis 3). Also, practitioners’ acceptance of AI is positively associated with trust, perception, and perceived risk (Hypotheses 4 to 6) but negatively associated with perceived risk (Hypothesis 7). The health care practitioners’ readiness to adopt AI in their practice is positively associated with acceptance (Hypothesis 8). The goodness-of-fit tests for the SEM are as follows: SRMR=0.0680, RMSEA=0.0913, GFI=0.8022, AGFI=0.7629, and Bentler CFI=0.9058. The SRMR value falls within the recommended level of less than 0.08. CFI is marginally above the acceptable level of 0.90. Although the GFI and AGFI values are below the recommended level of 0.85, and RMSEA is higher than the recommended level of 0.05, indicating moderate to mediocre overall fit of the proposed model to the observed data. The final SEM structure is shown in Figure 2.

**Table 7.** Structural paths for Trust, Perception, Risk, Benefit, Acceptance, and Readiness with standardized estimates and *P* values.

Path	Standardized estimate	Unstandardized parameter *P* value
Trust → acceptance	0.452	<.001
Risk → acceptance	−0.140	.009
Benefit → acceptance	0.168	.002
Perception → acceptance	0.459	<.001
Trust → perception	0.704	<.001
Trust → benefit	0.191	.02
Trust → *risk*	−0.301	<.001
Acceptance → readiness	0.874	<.001

## Discussion

### Overview

This exploratory, cross-sectional survey examined how Trust, Perception, perceived benefits, and perceived risks relate to Acceptance and Readiness for AI among United Arab Emirates–based health care practitioners. Trust reflects foundational beliefs about an AI system’s functionality, reliability, and helpfulness, and in our instrument spans propensity to trust technologies, understandability, technical competence, reliability, helpfulness, personal attachment, user autonomy, faith, and institutional credibility; conceptually, it precedes more specific evaluations. Perception summarizes a practitioner’s overall stance toward AI in practice, operationalized as perceived professional impact and preparedness for AI. Perceived risk and perceived benefit index respondents’ appraisal of potential harms versus advantages from AI use, treated as distinct, opposing evaluations. Acceptance aggregates intention-related and attitudinal components (eg, perceived ease of use or usefulness, attitude, social influence, perceived behavioral control, performance and effort expectancy, enjoyment, perceived fee, technicality, perceived value, behavioral, and purchase intentions), distinguishing it from the upstream belief constructs. Readiness differs from acceptance by focusing on actionable preparedness, knowledge, skills, and attitudes to apply AI in clinical care and is captured through cognition, ability, vision, and ethics. Finally, we recorded knowledge as both subjective awareness and objective (tested) knowledge for context and calibration, although the knowledge factor was not retained in the SEM.

### Principal Findings

One of the most significant findings is the central role of trust in AI technology. Trust’s positive link to perceived benefit and perception, coupled with its negative link to perceived risk, suggests that building institutional and technical trust in AI systems could accelerate its adoption among UAE health care professionals. The findings of this study align with prior research by Gefen et al [57], Karaca et al [51], and Mcknight et al [56], which positioned trust as an essential precursor in technology acceptance frameworks. Perception and benefit were positively and significantly associated with acceptance of AI, in line with existing literature indicating that when health care professionals perceive AI to be useful, accurate, and aligned with ethical standards, their willingness to accept its implementation increases [45]. Furthermore, risk showed a significant negative relationship with acceptance, confirming that concerns related to privacy, safety, and misuse can hinder adoption.

Standardized path coefficients from trust to perception are stronger than the paths from trust to risk and benefit, suggesting that fine-grained risk-benefit campaigns alone will yield limited gains unless accompanied by measures that raise global attitudes and institutional trust. In addition, trust holds both a sizable direct link to acceptance and sizable indirect links through perception, benefit, or risk. This pattern of relationships is coherent with early adoption contexts. Limited hands-on exposure to production AI systems likely dampens risk-benefit appraisals and elevates reliance on assurance cues (eg, reliability and accountability), which are captured in Trust. Programs that raise AI literacy alone may be insufficient unless they also build institutional or technical trust (eg, governance, transparency, or audits).

Another key insight is the significant relationship between acceptance and readiness, indicating that attitudinal intention precedes practical preparedness [29]. In our measurement, Acceptance (TAM) reflects willingness and intention shaped by usefulness or ease, norms, and control, whereas readiness (Medical Artificial Intelligence Readiness Scale) indexes whether clinicians possess the cognition, ability, vision, and ethics to act. The magnitude of this path suggests that when clinicians mentally commit to using AI, they are far more likely to report the skills, confidence, and situational preparedness needed to implement it. Practically, this means interventions that raise Acceptance (eg, governance transparency, clear accountability, and visible clinical benefit) can unlock and amplify the returns from skills training and workflow enablement. At the same time, acceptance is necessary but not sufficient: high intention without competency risks superficial adoption. These results, therefore, prioritize two-step programs; first, build acceptance through trust building and perceived value, and second, immediately pair that shift with competency-based training and supervised practice to consolidate readiness.

The “Knowledge” construct exhibited poor reliability (α=.14) and convergent validity (AVE=0.20) and was excluded from the SEM. These numbers are probably due to the absence of a prevalidated measure for AI literacy and the reliance on simple true or false statements to test the participants on AI facts. Therefore, the knowledge domain was assessed narratively based on descriptive statistics alone. Interestingly, although many participants expressed confidence in their knowledge of AI, objective assessments revealed notable knowledge gaps. This discrepancy between perceived and actual knowledge suggests the need for structured AI training programs that not only elevate baseline understanding but also address specific misconceptions. In addition, this gap suggests that self-ratings are insufficient proxies for deployable literacy. For training and policy, competency-based assessment (objective scoring and feedback) should accompany awareness sessions. Tailored professional development courses and the inclusion of AI modules in medical education could significantly enhance knowledge and confidence levels. Moreover, demographic analysis offers valuable nuance. The participant pool was diverse in terms of roles and years of experience, yet uniformly reflected limited prior AI training and inconsistent ability to recognize AI applications. These findings indicate that AI knowledge is not merely a function of professional role but is associated with broader systemic factors such as institutional support and exposure to technology in the workplace.

The clear path from trust to acceptance to readiness offers a practical roadmap for health care leaders and policymakers aiming to align AI adoption with national strategies. Aligned with the UAE’s AI Strategy 2031, we translate the trust-centered pathway into four linked levers. First, mandated, role-specific professional development, with tiered AI modules (foundation to specialty) and objective assessments. Second, governance checkpoints in procurement and deployment, audit trails, bias or risk communication, accountability mapping, and publicly available model factsheets to strengthen institutional trust. Third, usability and workflow fit through co-designed clinical pathways, pilot sites, supervised practice, and real-world feedback loops so that benefit and ease are evident at the point of care. And finally, readiness activation that converts acceptance into capability via hands-on credentialing (simulations and supervised cases) and change-management supports (local champions and help-desk service-level agreements).

### Comparison to Prior Work

Across studies from Saudi Arabia, the United Arab Emirates, Oman, and Egypt, health care professionals report moderate-to-high awareness of AI but limited practical experience and training, producing a persistent Knowledge-Readiness gap. For example, Almalki et al [70] found 76.6% high computer proficiency, alongside 62.1% low AI-specific knowledge, and only 20.6% of radiology professionals in Hamd et al [71] had received AI training. Attitudes are cautiously positive when AI is seen as useful and easy to use, for instance, 81% in Alhashmi et al [72], anticipated performance gains; nurses in Baraka et al [73] were receptive despite little prior use. Yet enthusiasm coexists with worries about job displacement, data confidentiality, reliability, and liability [74,75]. Barriers cluster into five repeatable categories: technical, professional, ethical, legal or regulatory, and resource, with technical and resource issues most frequently cited [71,73,76]. Facilitators mirror this profile with targeted education and curricular integration, organizational and IT infrastructure support, clear policy frameworks, and user-centered design being consistently linked to higher acceptance and readiness [72,73,77]. Acceptance also varies by demographic and professional factors; males, informatics-adjacent roles, technicians, and younger professionals tend to be more accepting, whereas senior physicians report lower familiarity and greater legal concerns [70,75,77,78].

This study’s central pattern aligns with findings across the Arab region studies that attitudes improve when AI is perceived as useful or easy and when organizational scaffolding is present. Our structural model clarifies a mechanism that prior surveys often imply but rarely quantify. We also replicate the well-noted Awareness-Readiness gap, that is, high subjective confidence coexists with uneven objective performance and limited formal training. Whereas most regional papers are cross-sectional and descriptive, our work advances the field by (1) reporting standardized path coefficients for all hypothesized relationships, (2) positioning trust as an upstream driver that reshapes perception and benefit and risk appraisals before linking to acceptance, and (3) explicitly quantifying the Acceptance-Readiness linkage rather than treating readiness as a descriptive end point. This moves the conversation from lists of correlates to a mechanism-oriented account of adoption. Moreover, our discussion echoes the reviewed literature corpus on the importance of readiness enablers that target the perceived usefulness and ease of AI use.

Set against prior work, our findings support a trust-centered adoption pathway; governance and organizational signals elevate trust, which shapes global perceptions more than a narrow risk-benefit tally; acceptance then gates implementation readiness. Regional studies document the same ingredients, but our modeling clarifies how these pieces combine and where interventions should focus: trust-building governance paired with role-aligned, competency-based training to convert positive attitudes into deployable practice.

### Limitations

First, like the broader literature, our sampling is constrained. Recruitment used purposive, network-based sampling, which precluded calculation of a response rate and prevented formal assessment of nonresponse bias; thus, selection effects may have favored more digitally engaged practitioners.

Second, although the sample size (N=182) is acceptable for exploratory SEM, it is modest relative to model complexity and limits power for subgroup comparisons and measurement-invariance testing.

Third, the generalizability of our findings is limited by the composition of the study sample. Participants were predominantly recruited from Dubai-based health care settings (103/182, 57%), and nurses constituted the largest professional group (71/182, 39%), which may bias observed relationships toward nursing workflows and the Dubai Health regulatory environment. Because health care governance, digital maturity, and implementation practices vary across Emirates and between federal and local systems, the results should not be interpreted as nationally representative of the entire UAE health care workforce. Accordingly, we interpret the SEM as an exploratory model validated within this cohort and recommend replication using larger, more representative samples (eg, stratified sampling across professions, care settings, and Emirates) before extending conclusions to the broader UAE health care workforce.

Fourth, convergent validity in some cases was marginally less than the acceptable threshold (AVE <0.50 for Perception and Risk); nevertheless, the factors were retained because their internal consistency and composite reliability were within the acceptable limits and their exclusion worsened overall SEM fit. However, the marginal psychometric strength of these constructs warrants cautious interpretation of pathways involving this construct and motivates replication with refined measurement in larger samples. Although the Acceptance-Readiness HTMT (0.848) is near the conservative cutoff, the value remains below the discriminant-validity threshold, indicating that Acceptance and Readiness are closely coupled but not empirically redundant, and future work should confirm this separation in larger samples and through additional validity checks.

Fifth, model fit could be improved with an increased sample size. Fit indices like the RMSEA, GFI, and AGFI all fall outside the acceptable range, indicating mediocre, suboptimal overall fit of the proposed model to the observed data. The fit indices were reported transparently to allow future work to build on this study. Our exploratory model should be validated, refined, and better established with improved fit.

Sixth, the Knowledge construct failed reliability or validity and was excluded from SEM, constraining conclusions about AI literacy. Any recommendations made regarding the knowledge gap came from the observed descriptive discrepancy as many respondents self-rated as informed, while objective item correctness was low for several items.

### Future Directions

Future work should prioritize upgrading the knowledge instrument. A validated, scenario-based scale with graded difficulty and objective scoring would better capture AI literacy. Reporting composite reliability and, where feasible, item-response metrics could establish performance benchmarks suitable for continuing professional development. Confirmatory testing with broader sampling is needed to strengthen external validity. Stratified or probability-based samples across Emirates, roles, and care settings, powered for multigroup and measurement-invariance tests (eg, nurses vs physicians and junior vs senior), should be paired with prespecified fit targets. Evaluation should move beyond attitudes to implementation outcomes. Adoption, fidelity, workflow time and interruptions, help-desk use, and safety signals (overrides and near-misses) ought to be tracked and linked to unit-level readiness. Human-centered workflow integration, co-designed pilots, electronic health record–embedded pathways, and iterative usability testing could define success via task completion time, error rates, and perceived workload. Finally, equity-focused targeting (eg, for professions or seniority groups with lower readiness) and transparent reporting of study instruments would facilitate replication and cumulative science.

### Conclusions

In the Arab-region literature, acceptance and readiness to implement AI depend on five interlocking domains: (1) knowledge or experience, (2) attitudes or perceived usefulness, (3) barriers (technical, professional, ethical, legal, and resource), (4) facilitators (education, organizational or IT support, policy frameworks, and usable design), and (5) demographic or professional influences. Current evidence suggests that positive attitudes need to be complemented with capability building and organizational or policy scaffolding; without them, the observed awareness does not translate into practice.

In this study cohort, trust and overall perception had stronger relationships with acceptance than specific benefit or risk appraisals, and acceptance was tightly linked to readiness. These results provide a mechanism-focused account of AI adoption in a UAE clinical cohort and identify governance- and training-related levers that can be operationalized in future, evaluative work. Replication with validated measures, broader sampling, and longitudinal designs is warranted.

## Supplementary material

10.2196/80173 Checklist 1 CHERRIES checklist.

## Abbreviations

AGFI: adjusted goodness-of-fit index
AI: artificial intelligence
AVE: average variance extracted
CFA: confirmatory factor analysis
CFI: comparative fit index
CHERRIES: Checklist for Reporting Results of Internet E-Surveys
CR: composite reliability
GFI: goodness-of-fit index
HTMT: heterotrait-monotrait ratio
RMSEA: root-mean-square error of approximation
SEM: structural equation modeling
SRMR: standardized root-mean-square residual
TAM: Technology Acceptance Model
TrAAIT: Theory of Trust and Acceptance of Artificial Intelligence Technology
UTAUT: Unified Theory of Acceptance and Use of Technology
