# Supplementary material for: Acceptance and Readiness for AI Among United Arab Emirates–Based Health Care Practitioners: Exploratory Cross-Sectional Survey
Source: JMIR AI. 2026 Apr 17;5:e80173. doi: 10.2196/80173 (PMC13097271; doi:10.2196/80173)
Supplement: Checklist 1 [file ai-v5-e80173-s001.pdf]

Checklist for Reporting Results of Internet E-Surveys (CHERRIES)

	**Checklist for Reporting Results of Internet E-Surveys (CHERRIES)**	
Item Category	***Checklist Item***	**Addressed in Paper?**
**Design**	Describe survey design	Addressed
**IRB (Institutional Review Board) approval and informed consent process**	IRB approval	Addressed
	Informed consent	Addressed
	Data protection	Addressed
**Development and pre-testing**	Development and testing	Addressed
**Recruitment process and description of the sample having access to the questionnaire**	Open survey versus closed survey	Addressed
	Contact mode	Addressed
	Advertising the survey	Addressed
**Survey administration**	Web/E-mail	Addressed
	Context	Addressed
	Mandatory/voluntary	Addressed
	Incentives	Addressed
	Time/Date	Addressed
	Randomization of items or questionnaires	Addressed
	Adaptive questioning	Addressed
	Number of Items	Addressed
	Number of screens (pages)	Not Addressed as it depends on device
	Completeness check	Addressed
	Review step	Not Addressed
**Response rates**	Unique site visitor	Addressed
	View rate (Ratio of unique survey visitors/unique site visitors)	Not applicable
	Participation rate (Ratio of unique visitors who agreed	Not applicable due to convenience sampling

	Checklist for Reporting Results of Internet E-Surveys (CHERRIES)	
Item Category	***Checklist Item***	**Addressed in Paper?**
	to participate/unique first survey page visitors)	
	Completion rate (Ratio of users who finished the survey/users who agreed to participate)	Not applicable due to convenience sampling
**Preventing multiple entries from the same individual**	Cookies used	Addressed
	IP check	Addressed
	Log file analysis	Addressed
	Registration	Addressed
**Analysis**	Handling of incomplete questionnaires	Addressed
	Questionnaires submitted with an atypical timestamp	
	Statistical correction	Addressed
